# Memory clinic experience under a social security health system in Costa Rica

**DOI:** 10.1590/S1980-57642014DN84000011

**Published:** 2014

**Authors:** Erick Miranda-Valverde, Daniel Valerio-Aguilar, Henri-Jacques Hernández-Gabarain, Cinthya Chaves-Araya, Monserrat Peralta-Azofeifa, Luis Emilio Corrales Campos, Rolando Angulo-Cruz, Ana Maricela Carballo-Alfaro, Alejandra Arias-Salazar, Silvia Araya-Segura, Fernando Morales-Martínez

**Affiliations:** Members of the Memory Clinic at the National Geriatrics and Gerontology Hospital, Dr. Raúl Blanco Cervantes, Costa Rica.

**Keywords:** Alzheimer, memory clinic, dementia, mild cognitive impairment, epidemiology, case register

## Abstract

Dementia and mild cognitive impairment (MCI) are an increasingly prevalent clinical entity in our field, showing an increasing incidence with age.

**Objective:**

The purpose of this study was to identify the main types of dementia and MCI treated in a memory disorders unit in Costa Rica.

**Methods:**

A consecutive and standardized register of patients diagnosed with dementia and MCI at the memory disorders unit of the National Geriatrics and Gerontology Hospital (NGGH) was analyzed.

**Results:**

Dementia was diagnosed in 63.5% of the 3572 cases, whereas 10.6% met criteria for MCI. The most frequent type of dementia was Alzheimer's disease (47.1%), followed by vascular pathology (28.9%), mixed forms (17.2%) and other types (6.8%). In MCI, 69.5% were of amnestic multiple domain type and 14.3% were non-amnestic multiple domain, while 41.3% were of vascular and 35.8% of neurodegenerative etiology. Mean age was 79.6±6.7 years and 64.7% were women in dementia cases whereas mean age was 76.4±6.9 years and 62.1% were women in MCI. Mean years of schooling was 4.95±4.09 years and 6.87±4.71, while mean time between onset of symptoms and clinical diagnosis was 3.2±2.6 years and 2.67±2.69 years, in dementia and MCI, respectively.

**Conclusion:**

The determination of the main types of dementia and MCI in Costa Rica and their main features has allowed the registration of comprehensive, hitherto unavailable information that will be useful for the management and strategic planning of public health care.

## Abstract (pt)

Demência e transtorno cognitivo leve (CCL) é uma entidade clínica cada vez mais prevalente, mostrando uma incidência crescente com a idade.

**Objetivo:**

O objetivo deste estudo foi o de identificar os principais tipos de demência e CCL tratados em uma unidade de transtornos da memória de Costa Rica.

**Métodos:**

Um registo consecutivo e padronizado de pacientes com diagnóstico de demência e CCL na unidade de transtorno de memória do Hospital Geral de Geriatria e Gerontologia.

**Resultados:**

Demência foi diagnosticada em 63,5% dos 3.572 casos, enquanto 10,6% tiveram diagnóstico de CCL. O tipo mais frequente de demência foi a doença de Alzheimer (47,1%), seguido de doença vascular (28,9%), formas mistas (17,2%) e outros tipos (6,8%). No CCL, 69,5% eram de tipo amnéstico de múltiplos domínios e 14,3% eram não-amnéstcios de múltiplos domínios, enquanto 41,3% eram de etiologia vascular e 35,8% de etiologia neurodegenerativa. A média de idade foi de 79,6±6,7 anos e 64,7% eram mulheres em casos de demência ao passo que a média de idade foi de 76,4±6,9 anos e 62,1% eram mulheres nos casos de CCL. A média de anos de estudo foi de 4,95±4,09 anos e 6,87±4,71, o tempo entre o início dos sintomas eo diagnóstico clínico médio foi de 3,2±2,6 anos e 2,67±2,69 anos em demência e CCL, respectivamente.

**Conclusão:**

A determinação dos principais tipos de demência e CCL na Costa Rica e as suas principais características permitiu o registro de informação abundante que era desconhecida e que será útil para a gestão e planejamento estratégico da saúde pública.

## INTRODUCTION

Alzheimer's disease (AD) and other dementias in developed countries have become one of the leading causes of death, with very high direct and indirect care costs. According to estimates, by 2050 the total number of such cases around the world is set to reach 115.4 million.^1^ In developing countries, the incidence of dementia is believed to be increasing, with Latin America estimated to lead in case growth by 2050. In most Latin-American countries, patients with dementia receive medical attention in private clinics or otherwise are offered partial subsidies.^2^ Even though a significant growth in people with dementia in this region of the American continent is expected, there is no data available for all countries in the region, including Central America.^3^ In the case of MCI, there is less data still. The National Geriatrics and Gerontology Hospital (NGGH) in San Jose, Costa Rica, is the only Memory Clinic in the country dedicated to the evaluation, diagnosis and treatment of memory disorders, MCI and dementia, operating under international protocols since February 2007.

The NGGH is a national specialized reference center for patients over 60 years of age who require comprehensive geriatric care. This hospital is part of the national social security system, which covers 95% of the Costa Rican population, and is financed with state funds and obligatory contribution by law from all those in active employment. Coverage includes medical attention for people of all ages and pregnant women, diagnostic tests and studies, surgical procedures, and medication for acute and chronic use.

The Memory Clinic is a multidisciplinary geriatric service consisting of two geriatricians, one neurologist, a psychiatrist, a clinical psychologist, two specialized nurses in Mental Health, a Pharmacist, and a Microbiologist. This unit is part of the hospital's outpatient department and is where patients complaining of memory problems are referred for evaluation and treatment. As it is a national referral center, patients from any area of the country can be treated. Costa Rica has an area of 51,100 square kilometers, divided into seven provinces. The population recorded by the National Institute of Statistics and Censuses for 2011 was 4,301,712 inhabitants, with an above 65 population of 311,712 inhabitants, representing 7.25% of the population.^4^

## METHODS

All patients were analyzed according to the same process of standardized assessment by evaluators from the Memory Clinic, which included: medical history and complete physical examination, application of the Mini-Mental State Examination (MMSE) test,^5^ the Clock Drawing Test when applicable, using the Cacho et al. scoring method,^6^ the Clinical Dementia Scale (Clinical Dementia Rating - CDR),^7^ the Barthel Rating Scale for activities of daily living,^8^ the Lawton rating scale for instrumental activities of daily living,^8^ the Yesavage Geriatric Depression Scale (GDS), 15-point version,^9^ the Cummings Neuropsychiatric Inventory (NPI)^10^ and the Reisberg Global Deterioration Scale (Global Deterioration Scale -GDS).^11^ In addition, a Neuropsychological Assessment was applied, with a score rating adjusted according to age and schooling -Neuropsi Test-Brief Neuropsychological Evaluation^12^ in cases with low education. In cases with high-school education, the Rey Auditory Verbal Learning Test was also used, Rey-Osterrieth complex figure test, phonetic and semantic verbal fluency, as well as the Trail Making Tests A and B. Other tests applied depended on the specificity of each case. In addition, all cases evaluated included complete blood count, kidney function tests, complete electrolytes, liver function tests, glucose, VDRL, thyroid function tests, Elisa human immunodeficiency virus (HIV), vitamin B 12 and folic acid. Similarly, all cases underwent neuroimaging study which consisted of a CT scan of the head without contrast medium. In those cases that so required, a structural brain MRI was performed. Initial evaluations were performed by two geriatricians, a neurologist and psychiatrist, all with expertise in the application and interpretation of the aforementioned tests as well as training in cognitive assessment, cognitive impairment and dementia. Neuropsychological assessments were performed by a Clinical Psychologist trained in neuropsychology. Once all cases completed the initial process, the final diagnosis was established in a Consensus Session with the participation of all the Memory Clinic members and based on current international diagnostic criteria for the different types of dementia and MCI.

## RESULTS

During the period spanning from the formation of the Memory Clinic to date, a total of 3572 patients with memory complaints were treated and evaluated, of which 63.5% met clinical criteria for dementia, 22.6% for mild cognitive impairment in some specific etiology and 10.6% were considered normal and/or with subjective memory complaints.

**Sociodemographic characteristics.** Mean age of the patients with MCI was 76.4±6.9 years and for dementia was 79.6±6.7 years (range 60-98 years) for both diagnoses, the majority aged between 70 and 89 years (87.1%) and 62.1% were women for MCI and 64.7% for dementia cases. Average schooling was 6.87±4.71for MCI and 4.95±4.09 years for dementia (range 0-28 years), 42.1% of cases were married in MCI cases and 37.2% in dementia, followed by widowhood at 40.7% for both diagnoses. The primary caregiver in MCI was the care-recipient's offspring in 34.1% and for dementia in 45.7% of cases.

**Clinical features.** In patients with dementia, mean time between onset of symptoms and clinical diagnosis was 3.18±2.6 years (range 0.0-25 years) and no differences were observed according to the subtype of dementia. The onset and course of symptoms was insidious in 76.9% and 67.3% of cases, respectively. A total of 27.1% of the cases were mild dementia, 31.9% moderate dementia, 39.3% moderate-severe dementia and 1.7% severe dementia according to the GDSc. According to the CDR result, 45.2% were mild dementia (CDR=1), 37.3% moderate dementia (CDR=2) and 17.6% severe dementia (CDR=3). The mean evolution of dementia according to severity was 2.6±2.1 years for mild forms, 3.2±2.6 years for moderate forms and 3.6±2.7 years for moderate-severe and severe forms (F=13.555, gl=2, p<0.001) according to the GDSc. Moreover, the mean evolution of dementia according to the CDR was 2.7±2.2for mild cases, 3.2±2.5for moderate cases and 4.2±3.1for severe cases. Regarding MCI cases, the mean time between onset of symptoms and clinical diagnosis was shorter than dementia, at 2.67 years, with a higher MMSE score of over 25 points and with a Clock Drawing Test score of 6.37 points. Functional outcome in MCI patients was greater than in dementia patients (Table 1).

**Table 1:** Clinical characteristics of patients according to dementia type and mild cognitive impairment. Memory Clinic NGGH 2007-2014.

	General	AD	VD	MD	Others	MCI
Symptom onset (y)	3.2±2.6	3.3±2.5	3.0±2.7	3.3±2.8	3.5±3.4	2.67±2.69
MMSE: mean±SD	18.35±5.9	18.4±5.6	18.7±6.2	17.97±5.9	18.65±5.4	2.6±3.42
Clock Test: mean±SD	3.4±2.5	3.5±2.4	3.5±2.7	4.29±2.5	3.12±2.69	6.37± 2.64
CDR Severity: mean±SD	1.72±0.74	1.76±0.72	1.59±0.74	1.76±0.76	1.83±0.84	0.46±0.18
Mild (CDR=1)	360 (45.2%)	149 (40.9%)	114 (55.6%)	57 (30.5%)	21 (44.7%)	NA
Moderate (CDR=2)	297 (37.3%)	151 (41.5%)	60 (29.3%)	48 (25.7%)	13 (27.7%)	NA
Severe (CDR=3)	140 (17.6%)	64 (17.6%)	31 (15.1%)	26 (13.9%)	13 (27.7%)	NA
GDS: mean±SD	3.18±3	2.95±2.77	3.39±3.22	3.17±3.33	4.29±3.63	3.64± 3.28
Barthel: mean±SD	84.3±2.8	87.45±17.26	79.94±23.95	2.59±20.4	81.34±24.93	94.5± 11.2
Lawton: mean±SD	2.8±2.3	3.06±2.29	2.60±2.25	4.29±3.63	2.56±2.27	6.05± 2.09
GDSc (most frequent category)	Moderate-severe	Moderate-severe	Moderate-severe	Moderate-severe	Moderate-severe	3

AD: Alzheimer’s disease; VD: Vascular Dementia; MD: Mixed Dementia; MCI: Mild Cognitive Impairment; MMSE: Mini-Mental State Examination; CDR: Clinical Dementia Rating; GDS: Geriatric Depression Scale; GDSc: Global Deterioration Scale.

Regarding dementia subtypes, Alzheimer's disease (probable or possible) was the most common, detected in 47.1% of cases (95%CI=43.6-49.7), followed by Vascular forms (probable or possible) in 28.9% (95%CI=27.5-33.1), mixed forms at 17.2% (95%CI=15.9-20.6) and other types of dementia at 6.8% (95%CI=5.3-11.3) (Table 2).

**Table 2:** Types of dementia registered. Memory Clinic NGGH 2007-2014.

Type dementia	# of cases	Percentage	95%CI
AD (probable / possible)	1003	47.1%	42.6-48.7
VD (probable / possible)	617	28.9%	27.5-33.1
Mixed forms	367	17.2%	15.9-20.6
Other	146	6.8%	10.3-14.3
Total	2133	100%	

AD: Alzheimer’s Disease; VD: Vascular Dementia.

Of the total cases for the Mixed Dementia category, 29.7% (95%CI=22.9-35.9) represented the combination of possible AD plus possible VD and 18.% (95%CI=13.1-24.3) for possible AD plus vitamin B 12 deficiency. Moreover, of all dementia cases from the Other types category, 9.52% (95%CI=4.4-14.6) represented Lewy Body Dementia and post-traumatic brain injury Dementia (Tables 3 and 4).

**Table 3:** Mixed forms of dementia. Memory Clinic NGGH 2007-2014.

Type of dementia	# of cases	Percentage	95%Ci
AD and VD possible	109	29.7%	22.9-35.9
AD possible and B 12 Def.	68	18.6%	13.1-24.3
AD possible and VD probable	59	16%	10.8-21.3
VD probable and B12 Def.	37	10.1%	5.8-14.5
Other various	94	25.6%	19.4-31.9
Total	367	100	

AD: Alzheimer’s Disease; VD: Vascular Dementia; B 12 Def: Vitamin B 12 deficiency; Other various: includes cases of multiple or rare combinations.

**Table 4:** Other forms of dementia. Memory Clinic NGGH 2007-2014.

Type of dementia	# of cases	Percentage	95%CI
Lewy Body Dementia	27	18.6%	4.4-14.6
Post TBI	30	20.6%	4.4-14.6
Dementia associated with Parkinson’s disease	21	14.5%	1.5-9.5
Dementia secondary to cerebral hypoxia	11	7.6%	1.5-9.5
Dementia from toxic metabolic origin	11	7.6%	1.04-8.5
Normotensive hydrocephalus	6	4.1%	0.11-6.2
Vitamin B12 / Folic Acid deficiencies	4	2.6%	–0.8-2.3
Dementia associated with PSP	4	2.6%	–0.8-2.3
Frontotemporal Lobar Degeneration / subtypes	4	2.6%	–0.8-2.3
Others	28	19.2%	
Total	146	100	

TBI: Traumatic Brain Injury; PSP: Progressive Supranuclear Palsy.

For patients diagnosed with MCI, almost 69.5% presented with a multiple domain and amnestic variant, meaning that there is impairment of at least two cognitive domains including memory, that is: language, calculation, orientation and/or executive functions (Table 5). Regarding the etiology of MCI, 41.3% were due to cerebrovascular disease, whereas 35.8% were neurodegenerative (Table 5).

**Table 5:** Types of MCI registered. Memory Clinic NGGH 2007-2014.

Type of Mild Cognitive Impairment	# of cases	Percentage	95%CI
Amnestic single domain	93	11.1%	7.9-14.3
Amnestic multiple domain	580	69.5%	64.8-74.2
Non-amnestic single domain	43	5.1%	2.9-7.4
Non-amnestic multiple domain	119	14.3%	10.8-17.9
Total	835	100	

## DISCUSSION

The information recorded above represents one of the first investigations of its kind in Costa Rica and Central America, allowing the identification of epidemiologic characteristics for dementia and MCI in our environment and comparisons with those obtained in other Latin American countries and the rest of the world.

During the investigation period, a total of 2346 dementia cases were recorded, of which 47.1% of cases were Alzheimer's disease related, followed by 28.9% secondary to vascular disease, which is consistent with data reported in other areas of the world and in Latin America, although the values differ slightly.^13,14^ Mixed forms represent the third most common type, where vascular pathology is often one of the main forms of combinations.^15^ This may occur because the recording procedure was done at a center which sees only patients over the age of 60, and therefore it is less likely to observe pure forms of a disease. Furthermore, we documented that the second form of primary degenerative dementia following Alzheimer's disease was Lewy Body Dementia, which is consistent with data reported in the literature. Lewy Body Dementia was present in 1.3% of cases, although this is slightly lower than the data reported in literature.^16^ The elevated incidence of vitamin B12 deficiency warrants attention in future research, and the possibility of mutations in enzymes that regulate the metabolism of this vitamin should be considered. The caregiver of most patients was their offspring or spouse, indicating that most cases are still household level care. The average time from onset of symptoms to diagnosis was 38.4 months (3.2 years), slightly higher than that reported in other published studies. This is an indicator that more education is required for the general^17^ population to seek professional care from the onset of early symptoms. Regarding the severity of dementia, 45.2% were mild forms, which again reinforces the fact that more information must be given to the general population to seek earlier care, and the remaining 54.6% were moderate or severe.

MCI cases of cerebrovascular etiology may be potentially preventable if there is adequate control of risk factors, such as high blood pressure, diabetes mellitus, lipid disorders, smoking and obesity. Moreover, controlling these risk factors may prevent conversion to vascular dementia. On the other hand, neurodegenerative MCI can evolve to Alzheimer's disease at an estimated rate of 16% per year, and some cognitive stimulation strategies, adequate control of cardiovascular risk factors, and physical activity may prevent this conversion. The NGGH has a program that includes cognitive stimulation for patients, and education for caregivers and family members.^18^

Diagnosing MCI provides an opportunity for specific interventions that may delay conversion to dementia, giving these patients more years of independence and functionality and a better quality of life.^19^

In conclusion, this report represents one of the first epidemiological reports on Dementia and MCI in Costa Rica, but is limited by the fact that only cases of patients over the age of 60 were recorded. Consequently, the behavior of presenile dementia and MCI remains unknown as these conditions are treated mostly in neurology services at other facilities. For this reason, it is necessary to encourage the creation of recording systems for early onset dementia in other centers. However, it is evident that the behavior of Dementia in Costa Rica is very similar to the pattern seen in the rest of the Western Hemisphere, while reports in Asian countries show different figures, with vascular forms as prevalent as neurodegenerative forms.^20-22^
